# TGC repeat expansion in the *TCF4* gene increases the risk of Fuchs’ endothelial corneal dystrophy in Australian cases

**DOI:** 10.1371/journal.pone.0183719

**Published:** 2017-08-23

**Authors:** Abraham Kuot, Alex W. Hewitt, Grant R. Snibson, Emmanuelle Souzeau, Richard Mills, Jamie E. Craig, Kathryn P. Burdon, Shiwani Sharma

**Affiliations:** 1 Department of Ophthalmology, College of Medicine and Public Health, Flinders University, Adelaide, South Australia, Australia; 2 Centre for Eye Research Australia, Royal Victorian Eye and Ear Hospital, Melbourne, Victoria, Australia; 3 Menzies Institute for Medical Research, University of Tasmania, Tasmania, Australia; University of Texas MD Anderson Cancer Center, UNITED STATES

## Abstract

Fuchs’ endothelial corneal dystrophy (FECD) is a progressive, vision impairing disease. Common single nucleotide polymorphisms (SNPs) and a trinucleotide repeat polymorphism, thymine-guanine-cytosine (TGC), in the *TCF4* gene have been associated with the risk of FECD in some populations. We previously reported association of SNPs in *TCF4* with FECD risk in the Australian population. The aim of this study was to determine whether TGC repeat polymorphism in *TCF4* is associated with FECD in the Australian population. In 189 unrelated Australian cases with advanced late-onset FECD and 183 matched controls, the TGC repeat polymorphism located in intron 3 of *TCF4* was genotyped using a short tandem repeat (STR) assay. The repeat length was verified by direct sequencing in selected homozygous carriers. We found significant association between the expanded TGC repeat (≥ 40 repeats) in *TCF4* and advanced FECD (*P* = 2.58 × 10^−22^; OR = 15.66 (95% CI: 7.79–31.49)). Genotypic analysis showed that 51% of cases (97) compared to 5% of controls (9) were heterozygous or homozygous for the expanded repeat allele. Furthermore, the repeat expansion showed stronger association than the most significantly associated SNP, rs613872, in *TCF4*, with the disease in the Australian cohort. This and haplotype analysis of both the polymorphisms suggest that considering both the polymorphisms together rather than either of the two alone would better predict susceptibility to FECD in the Australian population. This is the first study to report association of the TGC trinucleotide repeat expansion in *TCF4* with advanced FECD in the Australian population.

## Introduction

Fuchs’ endothelial corneal dystrophy (FECD, MIM 136800) is a progressive, degenerative disease of the corneal endothelium [1]. The clinical hallmarks of the disease include the presence of microscopic outgrowths (guttae), aberrant thickening of the Descemet’s membrane [2], and corneal endothelial cell loss [3]; Descemet’s membrane is the collagen rich basal lamina secreted by the corneal endothelium [4]. These pathological changes are accompanied by impaired ability of the corneal endothelium to pump excess fluid from the corneal stroma [1] which results in corneal oedema, pain, and ultimately vision loss, if left untreated [2]. Corneal transplantation, in the form of either penetrating or partial keratoplasty, is currently the only effective treatment for FECD [5].

The prevalence of FECD varies markedly across the world. In the USA, it affects ~4% of the population over the age of 40 [6] but is less frequent in Asian [7] and Middle-Eastern populations [8]. The prevalence has not been reported in Australia but corneal grafting for FECD accounted for ~26% (n = 395) of the corneal grafts (n = 1533) performed in 2014 [9], indicating that the disease is relatively common.

FECD is genetically a heterogeneous disease and manifests as two forms depending upon the age of onset. The rare, early-onset form [10] is typically inherited as an autosomal dominant disease with high penetrance and nearly uniform expressivity [2]. Mutations in the *COL8A2* (*collagen*, *type VIII*, *alpha 2*) gene account for some cases of early-onset FECD [11]; COL8A2, an extracellular matrix protein, is a major component of the Descemet’s membrane [12]. The more common late-onset FECD typically occurs after the age of 40 [13] and can be a familial disease; the risk of the disease increases with age and female sex [6]. Familial late-onset FECD shows an autosomal dominant inheritance with high penetrance but variable expressivity [6]. Mutations in the *ZEB1* (*zinc-finger E-box binding homeobox 1*), *SLC4A11* (*solute carrier family 4*, *sodium borate transporter*, *member 11*), *LOXHD1* (*lipoxygenase homology domains 1*) and *AGBL1* (*ATP/GTP binding protein-like 1*) genes cause the late-onset disease in a small number of familial, and/or unrelated cases [6, 8, 14–17]. Additionally, four chromosomal loci have been linked with familial late-onset disease but the causative genes are yet to be identified [17].

In 2010, Baratz and colleagues [18], through a genome-wide association study (GWAS) in white American cases, reported a highly significant association between single nucleotide polymorphisms (SNPs) in the *TCF4* (*Transcription factor 4*) gene and late-onset FECD; the intronic SNP rs613872 was the most significantly associated variant. Recently, our collaborative group through GWAS identified genome-wide significant association of SNPs in the *KANK4* (*KN motif- and ankyrin repeat domain-containing protein 4*) and *LAMC1* (*Laminin gamma-1*), and near *ATP1B1* (*Na+*, *K+ transporting ATPase*, *beta-1 polypeptide*) genes with FECD [19] and revealed three additional loci involved in the disease. However, the *TCF4* locus remains the strongest associated locus with FECD [19].

The *TCF4* gene is located on chromosome 18 and codes for the helix-loop-helix transcription factor E2-2 [18]. Our group, through an independent replication study, reported the association of SNPs in *TCF4* with advanced FECD in white Australians, which demonstrated the contribution of this gene to the disease risk in the Australian population [20]. Similar independent studies by other groups have shown association of SNPs, mainly rs613872, in *TCF4* with advanced FECD including in the Indian and Chinese populations [21–24]. Subsequently, Wieben and colleagues [25] identified a significant association of thymine-guanine-cytosine (TGC) repeat expansion in intron 3 of *TCF4* with FECD in white Americans and reported the repeat expansion to be a stronger predictor of the disease than SNP rs613872; repeat lengths of >50 were found to be more frequent in cases compared to controls [25]. The *TCF4* TGC trinucleotide repeat was first identified by Breschel et al. in 1997 and named as the CTG18.1 locus [26]. Association of this repeat expansion in *TCF4* with the disease has been replicated since in independent white American, Indian, Chinese and Japanese case cohorts [24, 27–30]; TGC repeat length of ≥40 was associated in these populations. This cut-off is based on the initial discovery that indicated that expansion of the TGC repeat locus in 3% of subjects in white pedigrees was not associated with any known phenotypes [26] whereas the expanded alleles with repeat lengths >37 were reported to be unstable [26]. Additionally, Mootha et al. [31] found segregation of the expanded TGC repeats in *TCF4* with the disease in several affected families with high or low penetrance indicating the importance of this repeat expansion in late-onset FECD. In this study, we aimed to determine whether TGC repeat polymorphism in *TCF4* is associated with late-onset FECD in the Australian population.

## Materials and methods

### Ethics statement, participant recruitment, and sample collection

The study was approved by the Southern Adelaide Clinical Human Research Ethics Committee, Southern Adelaide Local Health Network and Flinders University (South Australia, Australia), and the Human Research Ethics Committee of the Royal Victorian Eye and Ear Hospital (Melbourne, Victoria, Australia). The research was conducted in accordance with the guidelines of the National Health and Medical Research Council, Australia, and adhering to the tenets of the revised Declaration of Helsinki. All participants underwent a complete ophthalmic examination including slit lamp examination, confocal specular microscopy, and fundoscopy. Patients diagnosed with Grade 3–6 advanced late-onset FECD according to a modified Krachmer grading system [13] were recruited after obtaining written informed consent. Blood samples were collected from 189 participants through the Flinders Eye Clinic (Adelaide, South Australia, Australia) and the Royal Victorian Eye and Ear Hospital (Melbourne, Victoria, Australia). Control genomic DNA samples were from 183 unrelated, unaffected South Australian residents aged over 50 years recruited previously for use as controls in a variety of ocular genetic studies [32–34]. Genomic DNA from cases and controls was extracted using QIAamp DNA Blood Maxi kit (Qiagen Pty Ltd, Doncaster, Victoria, Australia) following the manufacturer’s protocol.

### STR assay and DNA sequencing

Gene-specific PCR primers (Forward: 5’-CAGATGAGTTTGGTGTAAGATG-3’, Reverse: 5’- ACAAGCAGAAAGGGGGCTGCAA-3’) used for amplification of the TGC trinucleotide repeat region in the third intron in the *TCF4* gene were the same as reported by Wieben et al [25]. A 5’FAM label was added to the forward primer. The TGC repeat polymorphism in genomic DNA was genotyped using short tandem repeat (STR) assay as previously described [25, 31]. Forty nanograms of genomic DNA was used as template and PCR was performed in a 10μl volume using HotStar Plus Taq® DNA polymerase (Qiagen GmbH, Hilden, Germany). The enzyme was activated at 95°C for 6 minutes followed by 35 cycles of denaturation at 95°C for 1 minute, annealing at 64°C for 1 minute, and elongation at 68°C for 3 minutes. The final elongation was at 68°C for 7 minutes. Each PCR product was diluted 1:10 and 1μl of the diluted product mixed with 0.15μl of GeneScan^TM^1200LIZ^®^ Dye Internal Size Standard (Applied Biosystems, Foster City, CA) and 8.85μl of Hidi Formamide (Applied Biosystems), and electrophoresed on a 3130xL Genetic Analyser (Applied Biosystems) according to standard protocols. The TGC repeat alleles were manually called using Peak Scanner^TM^ Software v1.0 (Applied Biosystems).

In selected cases and controls homozygous for the shortest or the longest repeat allele, Sanger sequencing was performed for validation of STR assay results. Amplification was performed as described above except unlabelled forward primer was used in a reaction volume of 20μl. The amplified products (5μl) were treated with 10U Exonuclease (New England Biolabs, Ipswich, MA, USA) and 2U Shrimp Alkaline Phosphatase (SAP; USB Corporation, Cleveland, Ohio, USA) at 37°C for 1 hour to digest and dephosphorylate remaining primer DNA and dNTPs; the reaction was terminated by incubation at 80°C for 20 minutes. Cleaned PCR products were sequenced using BigDye® Terminator v3.1 (Applied Biosystems) and forward primer, on a 3130xL Genetic Analyser (Applied Biosystems) according to standard protocols. DNA Sequences were aligned to the *TCF4* reference sequence (NM_001083962.1) retrieved from the Reference Human Genome sequence version 19 (hg19_RefGen), using the Sequencher^®^ software 5.0 (GeneCodes Corporation, Ann Arbor, MI). The TGC repeats were counted manually.

### Statistical analysis

Statistical analyses were performed using SPSS (Statistical Package for the Social Science; version 22) or in PLINK software [35]. Baseline characteristics of cases and controls–age and sex–were compared using Student’s t-test and chi-square test, respectively. The trinucleotide repeat alleles in cases and controls were compared by chi-square test. Difference in the distribution of the TGC repeat lengths between cases and controls was assessed by Mann-Whitney U test. A trend test for association of genotypes with FECD was performed in PLINK [35]. Logistic regression analysis was also performed in PLINK with age and sex as covariates to explore their effects. Conditional analysis was performed on SNP rs613872 (previously genotyped and association reported [20]) and the expanded TGC allele in *TCF4* to determine if the SNP was independently associated with the disease. Haplotype analysis was also performed using PLINK to investigate the effect of haplotypes of the associated *TCF4* expanded TGC repeat locus and the SNP rs613872, on FECD.

## Results

**Fig 1:**
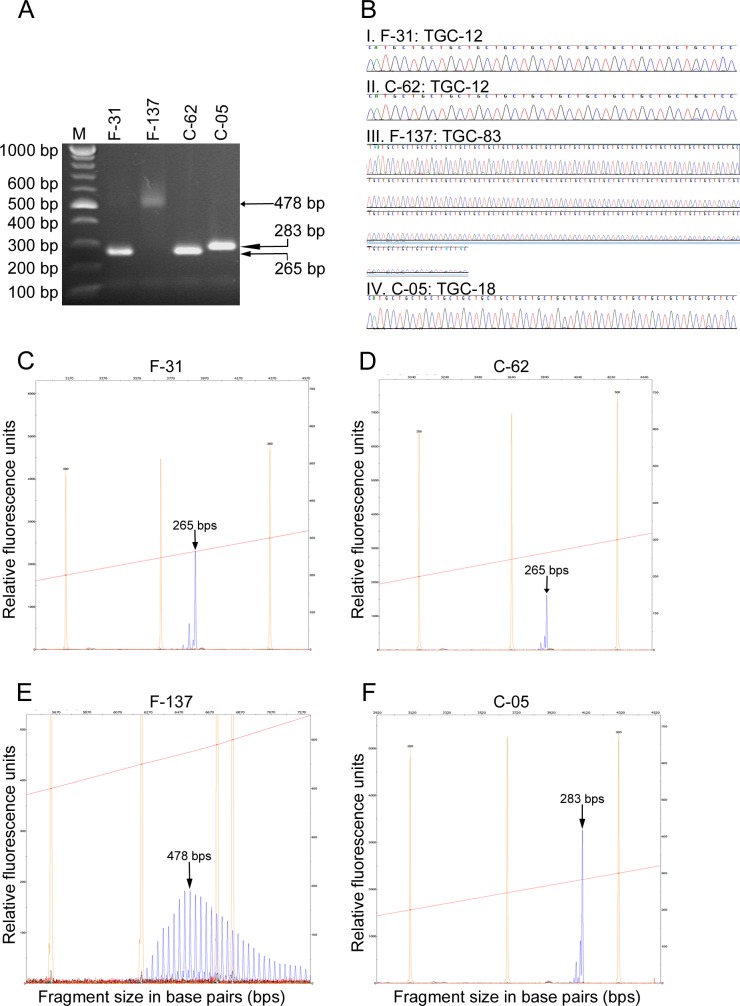
Analysis of *TCF4* TGC repeat polymorphism in homozygous FECD cases and controls. PCR was performed on genomic DNA from cases and controls carrying the shortest or the longest *TCF4* TGC repeat length. Data from two representative cases (F-31 and F-137 with 12 and 83 repeats, respectively) and controls (C-62 and C-05, with 12 and 18 repeats, respectively) are shown. **A.** Agarose gel electrophoresis of the PCR amplified repeat region from homozygous FECD cases and controls. Sizes of the products are shown on the right and correspond with the expected sizes (F-31 and C-62, 265 bp each; F-137, 478 bp; C-05, 283 bp). Sizes of DNA markers are indicated on the left. **B.** Sequencing chromatograms of FECD-affected (F-31 and F-137) and control (C-62 and C-05) individuals homozygous for the shortest (**B.I and B.II**) and the longest (**B.III and B.IV**) repeat alleles are shown. TGC repeat length in each individual was calculated by substracting 230 bps, corresponding to the DNA region flanking the repeat region amplified during PCR, from the detected PCR product size and dividing the difference by 3 (number of nucleotides of the repeat). **C-F.** Electropherograms showing the sizes of TGC repeat alleles in FECD cases F-31 (**C**) and F-137 (**E**) and in two control individuals C-62 **(D**) and C-05 (**F**) detected by STR assay. The peaks representing the *TCF4* TGC repeat fragments are indicated by arrows. Multiple peaks seen in panel E are due to variation in product size when large repeats are amplified. X-axis, fragment sizes in base pairs; Y-axis, relative fluorescence units; orange peaks, sizes of internal standards; red line across the electropherograms, slope threshold for peak start/end.

In this study, we determined an association between the trinucleotide repeat polymorphism in *TCF4* gene and FECD in white Australian cases by screening a total of 189 unrelated cases with advanced late-onset disease and 183 controls for the TGC repeat polymorphism in the gene. The TGC repeat was individually genotyped in each case and control by the previously reported STR assay [25]. The TGC repeats in selected homozygous cases and controls carrying the shortest repeat length (4 cases and 4 controls with 12 repeats each), and the longest repeat lengths (3 cases with 76, 83 and 84 repeats, respectively; 3 controls with 18 repeats each) were directly sequenced to confirm the repeat lengths. Size fractionation of the amplified repeat region showed products of the expected sizes in both cases and controls (Fig 1A) and confirmed the repeat lengths detected by the STR assay (Fig 1B). Sequence chromatograms and STR electropherogram traces of representative cases and controls are shown in Fig 1B–1F.

The characteristics of the case and control cohorts are given in Table 1. Females comprised 69% of cases and 72% of controls and the difference was not statistically significant (*p* = 0.555). A relatively higher percentage of females than males in the case cohort is consistent with the reported higher prevalence of FECD in females [36]. The controls were significantly older than cases (*p* = 0.023) by design to reduce the likelihood of yet to manifest disease.

**Table 1:** Characteristics of the FECD case and control cohorts, and dichotomised distribution of the *TCF4* TGC repeat alleles in cases and controls. The age and sex between cases and controls were compared using the Student’s t-test and chi-square test, respectively. Expanded allele counts between cases and controls were compared using chi-square test.

Description	Case	Control	*p*-value
N Participants	189	183	-
N Females (%)	130 (69%)	131 (72%)	0.555
Mean age in years ± SD	69.9 ± 11.2	76.6 ± 8.7	0.023
(Age range in years)	(32–93)	(42–96)	
N TGC repeat alleles with repeat length of <40/≥40	271/107	357/9	2.58 × 10^−22^

N = number; SD = standard deviation.

In accordance with denotation of the *TCF4* TGC repeat in previous studies [27, 31, 37], we dichotomised the TGC repeat alleles such that repeat length of ≥40 was considered as an expanded allele and <40 as a non-expanded allele. As shown in Table 1, the expanded allele is relatively rare in controls, but occurs on 28% of chromosomes in cases (*p* = 2.58 × 10^−22^; OR = 15.66 (95% CI: 7.79–31.49)). We found that cases carrying alleles with ≥40 TGC repeat lengths in *TCF4* are at 15 times greater risk of developing the disease than non-carriers. This is consistent with the findings of previous studies in white cases with FECD [25, 31], which also showed that those carrying expanded alleles of TGC repeats in this gene are at a greater risk of the disease than those carrying non-expanded alleles.

**Fig 2:**
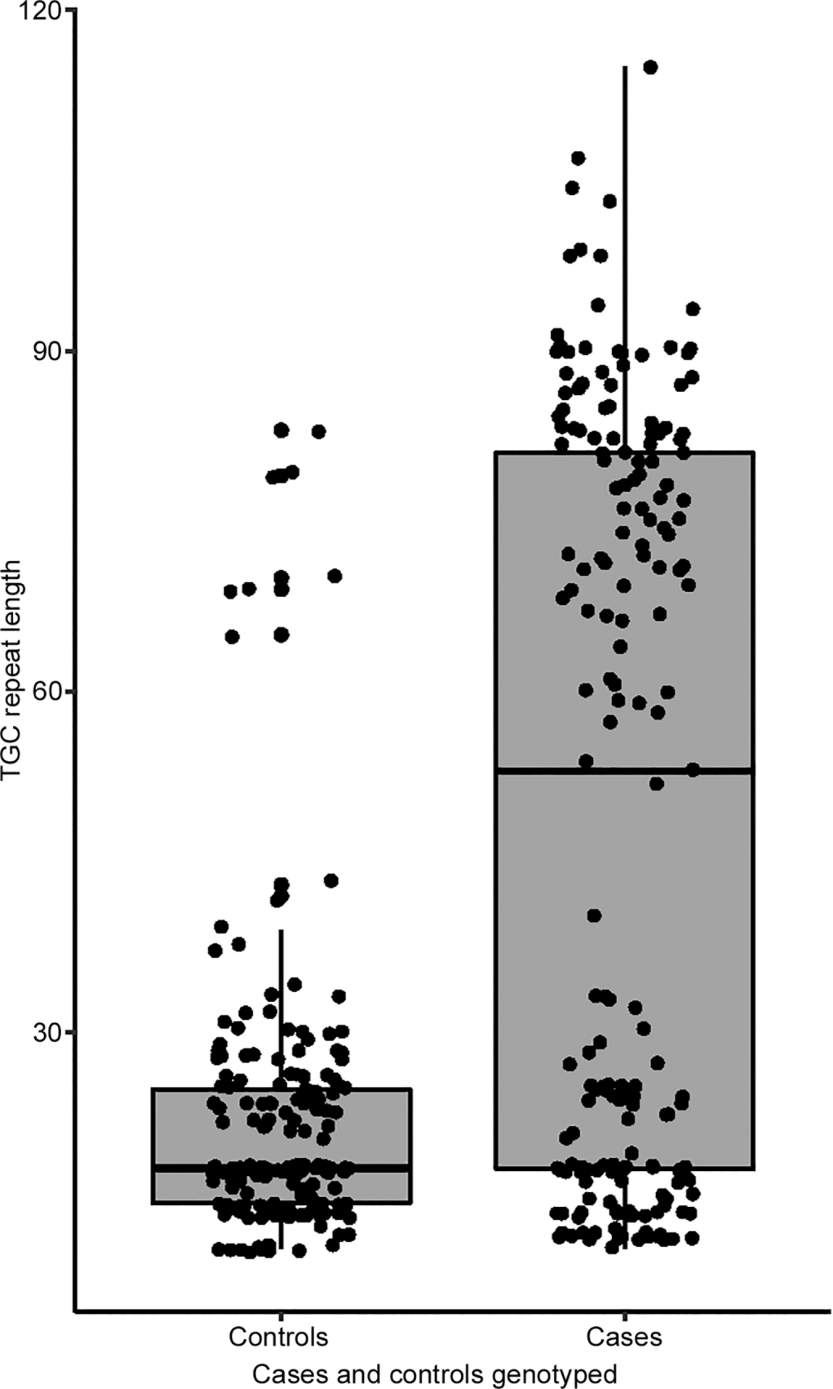
Distribution of TGC repeat lengths in the *TCF4* gene in FECD cases and controls. Median repeat length in cases = 53; range: 11–115, and median repeat length in controls = 18; range: 11–83. The box represents the second and third quartiles and the line in the middle indicates median. The lower and upper wiskers represent the limits of the first and fourth quartiles, respectively. The dots represent individual data points for controls (n = 183) and cases (n = 189).

Next, we assessed the distribution of the TGC repeat lengths in the study cohort. In cases, median repeat length was 53 and ranged from 11 to 115 whereas in controls it was 18 and ranged from 11 to 83 (Fig 2). The difference in the distribution of the repeat length in cases compared to controls was statistically significant (*p* = 0.0005).

**Fig 3:**
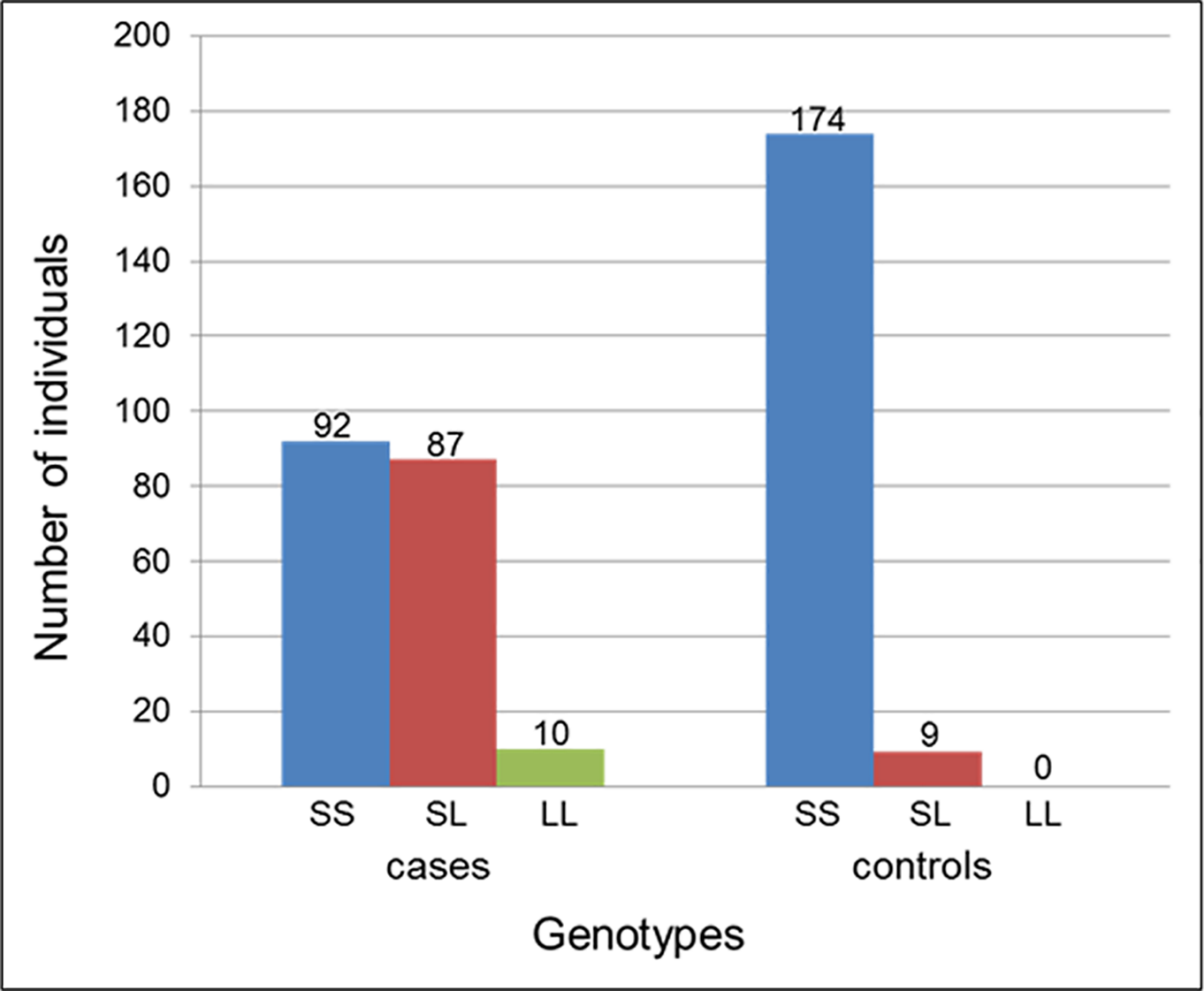
Distribution of genotypes of the *TCF4* TGC repeat alleles in FECD cases and controls. The numbers of individuals with each of the three possible genotypes of the dichotomised repeat alleles are shown. S represents short (<40 repeats; non-expanded) and L long (≥40 repeats; expanded) allele. SS represents homozygous non-expanded, LL homozygous expanded, and SL heterozygous with one non-expanded and one expanded allele.

Considering the genotype of the TGC repeat at the individual level, 87 cases (46%) were heterozygous and 10 cases (5%) homozygous for the expanded repeat allele; the remaining cases (n = 92; 49%) were homozygous for the non-expanded repeat allele (Fig 3). Of the cases homozygous for the expanded repeat, 7 cases carried both alleles with the same repeat lengths. Of those homozygous for the non-expanded repeat, 34 cases (18%) carried two alleles with different repeat lengths, and the remaining 58 cases (30.7%) carried both alleles with the same repeat lengths. In contrast, the majority of the controls were homozygous for the non-expanded repeat allele (n = 174; 95%) and a small proportion were heterozygous for the expanded repeat allele (n = 9; 5%). None of the controls carried the expanded repeat allele in a homozygous state (Fig 3). Of the homozygous non-expanded repeat carrying controls, the majority, 154 (84%), carried different repeat lengths on the two alleles; only 20 controls (11%) carried both alleles with the same repeat lengths. Overall, we observed a significant genotypic association between FECD and the expanded repeat allele (*p* = 3.92 × 10^−22^) in the Austalian population.

Next, we evaluated the effect of age and sex on the observed association through logistic regression analysis which revealed that the association of the expanded TGC repeat with the disease is independent of age and sex (*p* = 2.09 × 10^−14^; OR = 18.26 (95% CI: 8.67–38.46)). To determine any inter-dependence of association of the repeat expansion and the most associated SNP rs613872 in *TCF4* with FECD, we performed conditional analysis on the SNP. As evident from the reduced *p*-value and odds ratio, this analysis showed that association of the TGC repeat with the disease is partially independent of the SNP rs613872 (*p* = 9.74 × 10^−10^; OR = 10.76 (95% CI: 5.02–23.05)), and that using both polymorphisms together can better predict FECD susceptibility in Australian cases than either of the two alone. This was confirmed by the haplotype analysis of the associated *TCF4* TGC repeat locus and SNP rs613872, which revealed significant association with FECD with overall *p*-value of 7.74 × 10^−32^ (Table 2). A similar observation has been reported in non-Australian populations in an independent study [31].

**Table 2:** Association between common haplotypes of genotyped polymorphisms (TGC repeat locus, L/S, and rs613872, G/T) in the *TCF4* gene and FECD. f = frequency; OR = Odds ratio; CI = Confidence Interval; L = long repeat allele; S = short repeat allele.

	Over-all p-value = 7.74 × 10^−32^			
Haplotype	f cases	f controls	P-value	OR (95% CI)
LG	0.26	0.02	9.59 × 10^−24^	19.98 (9.08–44)
SG	0.21	0.14	0.009	1.68 (1.13–2.5)
LT	0.03	0.003	0.0002	113.3 (0.32–3.99× 10^4^)
ST	0.50	0.84	3.34 × 10^−26^	0.12 (0.08–0.19)

## Discussion

In this study, we determined the association between FECD and the expanded TGC repeat polymorphism in *TCF4* in Australian cases. The study reveals that the expanded TGC repeat is significantly associated with the disease in the white Australian population and is more significantly associated ((*P* = 2.58 × 10^−22^; OR = 15.66 (95% CI: 7.79–31.49)) than the rs613872 SNP ((*P* = 5.25 × 10^−15^; OR = 4.05 (95% CI: 2.82–5.83)) observed in our previous study [20]. Conditional analysis of the rs613872 SNP with the expanded TGC repeat suggests that these polymorphisms in *TCF4* are partially independently associated with FECD, implying that each can independently contribute to the pathogenesis of the disease. This study replicates the findings by other groups in American and Chinese cases with FECD [25, 27, 31]. It also suggests that considering both, the rs613872 SNP and the expanded TGC repeat polymorphism together can better predict susceptibility to FECD than either of the two alone, as shown by the haplotype analysis (*p*-value of 7.74 × 10^−32^, Table 2).

FECD is the first aging-related ocular disease to be associated with trinucleotide repeat expansion. Pathogenic expansion of trinucleotide repeat sequences has been reported in several neurodegenerative and neuromuscular diseases such as Friedreich’s ataxia and myotonic dystrophy type 1 [38–40]. Pathogenic expansion of both alleles of the GAA repeat present in the first intron of the *Frataxin* gene has been reported in the majority of cases with Friedreich’s ataxia; it contributes to the disease by prolonging transcription, resulting in a significant decrease in Frataxin protein levels [38, 41]. The disease can also occur in a minority of patients as a result of expansion of one GAA repeat allele and the presence of a point mutation in the second allele [41]. Regardless of the genetic abnormality, transcription inhibition causes loss-of-function of *Frataxin*, and consequently pathogenesis of Friedreich ataxia [42]. The FECD-associated expanded TGC repeat in *TCF4* is also an intronic repeat. However, our differential gene expression analysis data (unpublished) and reported studies [28, 43] have shown that expression levels of *TCF4* mRNA in the corneal endothelium between FECD cases and controls and between carriers of risk and non-risk alleles of SNP rs613872 are similar indicating that transcription inhibition likely is not involved in the pathogenesis of FECD.

RNA-mediated gain-of-function mechanism has been shown to underlie myotonic dystrophy type 1 [38] that is caused by expansion of the CTG repeat in the 3’UTR of the *DMPK* (*Dystrophia myotonica protein kinase*) gene [38]. The expanded CTG repeat has been reported to affect alternative splicing of the *DMPK* gene [38, 44]. The expanded DMPK (CUG)_n,_ mRNA is transcribed normally but fails to undergo translation and is thus retained in the nucleus as hairpin structures [45–47]. The hairpin structures exhibit a toxic dominant gain-of-function abnormality by sequestration and accumulation of RNA-binding regulatory proteins, such as the alternative splicing regulator muscle blind-like 1 (MBNL1) and CUG triplet repeat RNA-binding protein 1 (CUG-BP1) [48], and formation of nuclear foci [49–53]. The aberrant nuclear foci cause cellular toxicity that contributes to the disease pathogenesis [44]. Interestingly, Mootha et al. [28] and Du et al. [54] reported the presence of RNA nuclear foci in a proportion of corneal endothelial cells in some patients with FECD carrying the *TCF4* expanded TGC repeat. The expanded mRNA co-localised with sequestered MBNL1 protein in nuclear foci leading to mis-splicing of MBNL1-regulated transcripts. These reports implicated RNA toxicity and mis-splicing in the pathogenesis of FECD, and possibly a shared disease mechanism between FECD and myotonic dystrophy type 1 disease [28, 54]. The recent study by Wieben et al [55] found mis-splicing of several genes in the corneal endothelium of FECD cases carrying expanded TGC repeat in *TCF4*, and validates these findings [28, 54]. Thus, RNA mis-splicing induced by TGC trinucleotide repeat expansion in the *TCF4* gene is the likely mechanism of pathogenesis in patients with FECD carrying the expanded repeats [55]. However other yet to be identified mechanisms may underlie the disease in patients carrying non-expanded repeats in this gene.

The STR analysis employed for detection of TGC repeat lengths in this study, is unable to detect very large repeat expansions of several hundred repeats, which is a limitation of the study. Previous studies have reported the prevalence of very large TGC repeat lengths in *TCF4* in ~5% of FECD cases [37]. Assuming similar prevalence of very large repeat expansions in the Australian cohort, nine cases, and one of the 20 controls found to carry the same non-expanded repeat length on both the alleles may carry a very large repeat expansion that was not detected in this study. This missing information would further strengthen the overall findings of the study.

In conclusion, this study provides independent evidence for the association of the expanded TGC repeat polymorphism in the *TCF4* gene with advanced FECD. This is the first study to report an association of the repeat polymorphism in *TCF4* with FECD in the Australian population and further confirms the contribution of this gene to FECD susceptibility in Australia. The findings suggest a partially independent association of the expanded TGC repeat and SNP rs613872 in *TCF4* with FECD in Australian cases, and indicate that considering both the polymorphisms together would better predict susceptibility to FECD in the Australian population.
